# Supplementary material for: Emergent microrobotic oscillators via asymmetry-induced order
Source: Nat Commun. 2022 Oct 13;13:5734. doi: 10.1038/s41467-022-33396-5 (PMC9561614; doi:10.1038/s41467-022-33396-5)
Supplement: Supplementary file 1 — Supplementary Information [file 41467_2022_33396_MOESM1_ESM.pdf]

# Emergent microrobotic oscillators via asymmetry-induced order

Supplementary Information

**Jing Fan Yang**[*1] **Thomas A. Berrueta**[*2] **Allan M. Brooks**[1] **Albert Tianxiang Liu**[1,3] **Ge Zhang**[1] **David Gonzalez-Medrano**[4] **Sungyun Yang**[1] **Volodymyr B. Koman**[1] **Pavel Chvykov**[5] **Lexy N. LeMar**[1] **Marc Z. Miskin**[4] **Todd D. Murphey**[2] **Michael S. Strano**[†1]

[1]Department of Chemical Engineering, Massachusetts Institute of Technology, Cambridge, MA, USA.
[2]Center for Robotics and Biosystems, Northwestern University, Evanston, IL, USA.
[3]Department of Chemical Engineering, University of Michigan, Ann Arbor, MI, USA.
[4]Department of Electrical and Systems Engineering, University of Pennsylvania, Philadelphia, PA, USA.
[5]Physics of Living Systems, Massachusetts Institute of Technology, Cambridge, MA, USA.

## Contents

**Supplementary Notes** **2**
1 Mechanistic Model and Simulation of Particle Beating . . . 2
2 Thermodynamics of Particle Beating . . . 5
3 Note on Microelectronic Low-Frequency Oscillators . . . 10
4 Note on the Fuel Cell's Open-Circuit Voltage . . . 10
5 Note on the Energy Expenditure . . . 11
5.1 Energy Conversions of the Mechanical Oscillation . . . 11
5.2 Energy Conversions of the Microgenerators . . . 11

**Supplementary Figures** **12**

**Supplementary References** **20**

[*]These authors contributed equally to the work.
[†]Corresponding author: strano@mit.edu

# Supplementary Notes

## 1 Mechanistic Model and Simulation of Particle Beating

As the microparticles beat at the curved liquid-air interface of a drop of aqueous $H_2O_2$ solution, we start by solving the Laplace equation of capillarity [1, 2]. We solved the following system of ordinary differential equations (ODEs) with MATLAB's ode45 Runge-Kutta solver (MathWorks, Inc., Natick, MA):

$$\frac{d\rho}{ds} = \cos\theta \tag{1}$$

$$\frac{dz}{ds} = \sin\theta \tag{2}$$

$$\frac{d\theta}{ds} = \begin{cases} \beta, \; s = 0 \\ 2\beta + \gamma_c z - \dfrac{\sin\theta}{\rho}, \; s > 0 \end{cases} \tag{3}$$

$$\frac{dV}{ds} = \pi\rho^2 \sin\theta \tag{4}$$

$$\rho(0) = z(0) = \theta(0) = V(0) = 0 \tag{5}$$

where $\beta$ is the curvature at the apex $s = 0$ and $V$ is the volume. $\gamma_c = g(\rho_l - \rho_a)/\gamma$ denotes the capillary constant, where $\gamma$ is the interfacial tension, $\rho_l$ density of the liquid phase, and $\rho_a$ that of air. $\theta$, $r$, $z$, and $s$ are defined in Supplementary Fig. 1. For each value of $\beta$, a solution to the initial value problem can be obtained which describes the profile of a Laplacian axisymmetric interface. A unique $\beta$ can be identified such that $V = V_{drop}$ and $\theta = \theta_c$ at the three-phase contact line. In our experimental system, the contact angle $\theta_c = 87.4°$ for the peroxide-polystyrene interface in air. Supplementary Fig. 1 below presents the interface profile for a series of $V_{drop}$ values. $\beta$ is solved to be $17.56 \text{m}^{-1}$ for $V_{drop} = 1\text{mL}$.

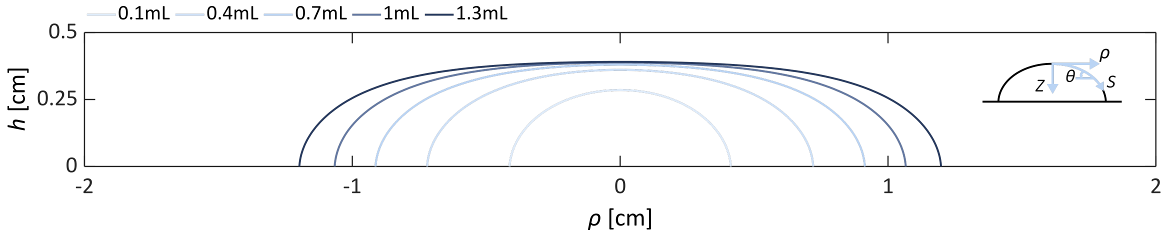


Supplementary Figure 1: Coordinate system of the $H_2O_2$ drop and the solved interface profiles for a series of drop volumes $V_{drop}$.

As discussed in the main text, the microparticles are driven outward by the collapse of a shared bubble and come together via a global and a local restorational force. As the SU-8 polymer is barely denser than the peroxide solution, the buoyancy from a small gas bubble underneath the disc is able to overcome the particle's weight and create a net force upward. As the microparticle is constrained to the liquid-air interface, it climbs the global drop profile defined by the above solution of the Laplace equation. One can formulate the energy as the product of the particle's vertical displacement and its weight after the subtraction of the Archimedes force. Thus, the lateral component of this global restorational force is given by:

$$F_g = [m - \rho_l \Lambda_b V_b(t)]\, g \frac{dz}{d\rho} \tag{6}$$

where $V_b(t)$ is the instantaneous bubble volume. Only the mass of the particle, $m$, is considered as that of the bubble is insignificant. The dimensionless factor $\Lambda_b$ is the volume fraction of the gas bubble lying below the undisturbed interface, as it is the displaced liquid in this region that gives rise to buoyancy. Note that we only included $\Lambda_b$ for the generality of Supplementary Equation (6). We use a $\Lambda_b$ of unity in the simulations hereafter in accordance with experimental observations. Given the drop profile, the force always points towards the apex.

To quantify the "Cheerios effect", i.e. the inter-particle capillary attraction as a result of the local interfacial distortion, we adopt the Nicolson approximation [3] which assumes that (i) the horizontal force from capillary pressure is insignificant compared to that from buoyancy, and (ii) the small interfacial distortions may be superposed [4]. Prior results show that the Nicolson approximation is justified for small Bond numbers $B = R^2/L_c^2$, or equivalently if the floating object's radius $R \ll L_c = \sqrt{\gamma/\rho_l g}$, the capillary length. Indeed, the $L_c$ of our experimental system is approximately 2.7mm, far exceeding the spatial scale of the beating physics. The surface height in the neighbourhood of a floating bubble follows:

$$h(l) = -B\Sigma R K_0(l/L_c) \tag{7}$$

where $l$ is the lateral distance from the bubble centre, $K_n$ the modified Bessel function of the second kind of order $n$, and $\varSigma$ the buoyancy-corrected dimensionless weight defined by $2\pi\gamma RB\varSigma = [m - \rho_l \varLambda_b V_b(t)]\, g$. Supplementary Equation (7) is a simplified asymptotic result true for $l \ll L_c$. The lateral capillary force experienced by a bubble of volume $V_b'$ at a distance $l$ away is therefore:

$$F_c = [m - \rho_l \varLambda_b V_b'] \, g B^{3/2} \varSigma K_1(l/L_c) \tag{8}$$

Needless to say, the capillary attraction force points towards the centre of the other particle. The $K_1(l/L_c)$ dependence is in agreement with the results derived from an energy approach [5]. Readers are directed to [6] for the treatment of scenarios with more than 2 particles.

We next consider the hydrodynamic interactions. In the regime of low Reynolds number and low capillary number such as our system, the drag force for an object at the liquid-air interface is expressed as:

$$\mathbf{F}_d = -6\pi\mu\varLambda_d R_b(t)\mathbf{v}(t) \tag{9}$$

where $\mu$ is the liquid's dynamic viscosity, $R_b$ the bubble radius, and $\mathbf{v}$ the instantaneous velocity. The drag coefficient $\varLambda_d$ is a scaling factor depending on the object's geometry, its depth of immersion, the contact angle, surface tension, and the densities of the object and the liquid [6]. As $\varLambda_d$ is difficult to estimate analytically, we assume it is a constant for simplicity and leave it as one of the two free parameters we estimate from experiments, a practice consistent with published models of microparticle motion along a curved interface [2].

An important additional consideration is the significantly increased drag when multiple particles approach one another, caused by the increased resistance to removing the liquid between them [7]. We note this inter-particle hydrodynamic interaction particularly because of the noticeable deceleration in our beating system when the edge-to-edge distance between particles were less than $2R_p$ (see, for example, Fig. 1g between 68 and 69s). The approach velocity was virtually 0 right before contact, suggesting a drag significantly larger than that given by Supplementary Equation (9). Indeed, some previous studies predicted two floating microparticles to accelerate towards each other all the way until they collide if the Stokes' drag expression was not corrected for inter-particle interactions [4].

The most numerically convenient means of accounting for said interactions is to adopt the concept of hydrodynamic mobility [8], as with a number of previous works [6, 9]. This correction factor as a function of the inter-particle spacing, $l$, is given by:

$$G(\lambda) = 1 - \frac{1}{3}\lambda^{-1} + \lambda^{-3} - \frac{15}{4}\lambda^{-4} - \frac{4.46}{1000}(\lambda - 1.7)^{-2.867} \tag{10}$$

where $\lambda = l/\max[R_b(t), R_p]$. $G$, which is typically multiplied to the terminal velocity, approaches 1 for large separations $\lambda \to \infty$ and 0 for $\lambda = 2$ when the objects contact. Equivalently, we divided the drag expression in Supplementary Equation (9) with $G$ in our numerical simulations.

The force expressions in Supplementary Equations (6), (8), (9), and (10) allow us to simulate the motion of each beating particle $i$ with Newton's second law:

$$\frac{d\mathbf{v}_i}{dt} = \frac{1}{m_{\text{eff}}}(\mathbf{F}_{g,i} + \mathbf{F}_{c,i} + \mathbf{F}_{d,i}) \tag{11}$$

We followed [2] in introducing a scaling factor for the effective mass ($m_{\text{eff}} = \varLambda_m m$) to account for the added mass of liquid experienced during particle acceleration. The two fitted parameters of the model, $\varLambda_m = 11.25$ and $\varLambda_d = 0.35$, were kept constant across simulations of different $H_2O_2$ concentrations. The model outlined above was solved numerically again with MATLAB's ode45 Runge-Kutta solver. As all three forces are also dependent on the instantaneous bubble volume (equivalently, the radius), we zoom in to the catalytic surface and study the reaction kinetics as the final piece of the puzzle.

The volume of the $O_2$ bubble as a function of time, $V_{b(t)}$, is dictated by the rate of $O_2$ generation, which in turn is dependent on the free platinum patch surface area $A_{\text{Pt,free}}$, as well as the peroxide concentration $[H_2O_2]$. For a given experiment, we assume that the peroxide is in excess and $[H_2O_2]$ is a constant throughout, based on the absence of a shift in the beating frequency (Supplementary Fig. 6). A well-studied catalytic reaction, the decomposition kinetics of $H_2O_2$ on noble metal and oxide surfaces can be described by the classic Langmuir-Hinshelwood mechanism [10, 11]:

$$\frac{dV_b}{dt} = \frac{kA_{\text{Pt,free}}(V_b)[H_2O_2]}{1 + K_H[H_2O_2]} \tag{12}$$

where $k$ is a constant encompassing the reaction rate constant, the specific volume of $O_2$, and the areal density of the surface sites. The kinetic equation represents that the rate is first order with respect to the concentration of bound surface sites, which are saturated at increasing peroxide concentration modulated by the binding constant $K_H$. In the single particle scenario, $A_{\text{Pt,free}}$ decreases over time as the bubble underneath the particle starts to limit the accessible catalytic surface area. This leads to a reduced $dV_b/dt$ and therefore a self-limiting reaction. Inspection of the 2-particle beating videos, on the other hand, shows a near-linear increase of the bubble volume up until the moment of merger. This observation

suggests that the bubbles in the beating system do not grow beyond the critical $V_\mathrm{b}$ which marks the onset of catalytic surface blockage, and that $A_\mathrm{Pt,free} \approx A_\mathrm{Pt}$. The resultant time-independent reaction rates at different $H_2O_2$ molarities were fitted to the Langmuir-Hinshelwood kinetics, outputting $k = 3.025 \times 10^{-10}\mathrm{m^4 s^{-1} mol^{-1}}$ and $K_\mathrm{H} = 0.677\mathrm{L/mol}$. This parameterised kinetics was used in our quantitative model to account for the effects of $H_2O_2$ concentration and Pt surface area (e.g. in Fig. 1j of the main text).

All parameters used in the mechanistic model are listed in Supplementary Table 1.

Supplementary Table 1: **Parameters used in the mechanistic model.**

| Symbol | Parameter | Conventional Unit |
|---|---|---|
| $\beta$ | Curvature at the apex of the drop | [$\mathrm{m^{-1}}$] |
| $\gamma$ | Interfacial tension of the liquid-air interface | [N/m] |
| $\gamma_\mathrm{c}$ | Capillary constant of the liquid drop | [$\mathrm{m^{-2}}$] |
| $\theta$ | Angle of the liquid-air interface relative to the lateral dimension | [rad] |
| $\theta_\mathrm{c}$ | Three phase contact angle | [rad] |
| $\lambda$ | Dimensionless distance between two floating objects used in hydrodynamic mobility | [-] |
| $\Lambda_\mathrm{b}$ | Volume fraction of gas bubble lying below the undisturbed interface | [-] |
| $\Lambda_\mathrm{d}$ | Effective drag scaling factor | [-] |
| $\Lambda_\mathrm{m}$ | Effective mass scaling factor | [-] |
| $\mu$ | Dynamic viscosity of the liquid | [$\mathrm{N{\cdot}s{\cdot}m^{-2}}$] |
| $\rho$ | Lateral coordinate relative to the drop's apex | [m] |
| $\rho_\mathrm{a}$ | Density of air | [$\mathrm{kg/m^3}$] |
| $\rho_\mathrm{l}$ | Density of liquid | [$\mathrm{kg/m^3}$] |
| $\Sigma$ | Buoyancy-corrected dimensionless weight | [-] |
| $A_\mathrm{Pt}$ | Surface area of the Pt patch | [$\mathrm{m^2}$] |
| $A_\mathrm{Pt,free}$ | Accessible area of the Pt patch | [$\mathrm{m^2}$] |
| $B$ | Bond number | [-] |
| $F_\mathrm{c}$ | Lateral component of the local capillary force between floating objects | [N] |
| $F_\mathrm{d}$ | Lateral component of the drag force | [N] |
| $F_\mathrm{g}$ | Lateral component of the global buoyancy-corrected gravitational force towards the apex | [N] |
| $g$ | Gravitational acceleration | [$\mathrm{m/s^2}$] |
| $G$ | Hydrodynamic mobility | [-] |
| $[H_2O_2]$ | Concentration of hydrogen peroxide | [mol/L] |
| $k$ | Rate constant of peroxide decomposition per unit area | [$\mathrm{m^4{\cdot}s^{-1}{\cdot}mol^{-1}}$] |
| $K_\mathrm{H}$ | Surface binding constant of peroxide decomposition | [L/mol] |
| $K_n$ | Modified Bessel function of the second kind of order $n$ | [-] |
| $l$ | Lateral coordinate from the centre of a floating object | [m] |
| $L_\mathrm{c}$ | Capillary length | [m] |
| $m$ | Mass of a microparticle | [kg] |
| $m_\mathrm{eff}$ | Effective mass of a microparticle | [kg] |
| $R$ | Radius of a floating object | [m] |
| $R_\mathrm{b}$ | Instantaneous radius of a bubble | [m] |
| $s$ | Coordinate along the liquid-air interface relative to the drop's apex | [m] |
| $t$ | Time | [s] |
| $v$ | Lateral component of a microparticle's velocity | [m/s] |
| $V$ | Integral volume used in calculating the drop profile | [$\mathrm{m^3}$] |
| $V_\mathrm{b}$, $V'_\mathrm{b}$ | Volume of a bubble | [$\mathrm{m^3}$] |
| $V_\mathrm{drop}$ | Volume of the drop | [$\mathrm{m^3}$] |
| $z$ | Vertical coordinate relative to the drop's apex | [m] |

## 2 Thermodynamics of Particle Beating

Asymmetry-induced order is a process by which explicit symmetry-breaking (spontaneous or otherwise) leads to the emergence of ordered states in a system [12–15]. Hence, asymmetry-induced order requires both a symmetry whose breaking can be observed, and a clear notion of "degree of order." Which symmetry to break is inherently a system-dependent question, and as such there are no general means of choosing between symmetry groups to achieve a desired outcome. However, the so-called degree of order of a system is a challenging property to formally specify in general. For one, what is meant by order is often ill-defined or underspecified. Secondly, even when provided with a means to metricize order, such metrics are often analytically and computationally intractable because they require global knowledge of system states—as is the case for calculating entropy. This is further complicated by the fact that, far from equilibrium, entropy is not sufficient to establish the robustness, stability, or persistence of system configurations (all of which are attributes often ascribed to "orderly" states) [16]. To this end, physicists have made use of order parameters to establish more narrowly-construed notions of order on a case-by-case basis for particular systems [17, 18].

Recent work in nonequilibrium thermodynamics has made strides towards describing the emergence of order more generally in broader classes of complex systems. Rattling theory is a novel thermodynamic theory describing the emergence of order and self-organization in "messy" nonequilibrium dynamical systems [19, 20]. The success of rattling theory depends crucially on the definition of the class of systems it considers to be messy. The rattling ansatz sees the behavior of complex systems as stochastic diffusion processes taking place in high-dimensional configuration spaces in the presence of energy influxes driving them out of equilibrium. Any system whose behavior can be described by such configuration-space diffusion falls under the class of messy systems described by rattling theory. Modelling the behavior of systems as diffusion processes is what enables an analytical determination of nonequilibrium steady-state density, and, as a consequence, an understanding of self-organization. Empirically, this approach has been shown to predict the long-term behavior of a wide variety of systems, from canonical chaotic systems [21] to swarms of robots [19], and is expected to apply across diverse active matter systems as well [22, 23].

At the heart of the theory lies a precise, local, and computable measure of order, *rattling*, from which the theory derives its name. Rattling measures the way in which system configurations respond to external force fluctuations: Rapid, uncorrelated configurational changes produce high rattling values, and slow, correlated changes produce low rattling values. When a system's response to local force fluctuations is random (i.e., has Gaussian statistics), rattling is exactly the entropy of its configurational velocities. As a quantity, the rattling $\mathcal{R}(q)$ of a system at configuration $q$ is

$$\mathcal{R}(q) = \frac{1}{2} \log \det \langle \dot{q}_i(t), \dot{q}_j(t) \rangle_{q(0)=q} \tag{13}$$

where $\langle \cdot, \cdot \rangle$ is the covariance tensor of the system's configurational velocities (i.e., two-point correlation function) averaged over an ensemble of dynamical trajectories initialized at $q$. Using this definition, we can state the central prediction of rattling theory, known as the "low-rattling selection principle." The principle expresses a relationship between the magnitude of system-level fluctuations measured at a particular configuration (i.e., rattling $\mathcal{R}(q)$), and said configuration's prevalence in the system's nonequilibrium steady-state density. In particular, this relationship is of Boltzmann-like form:

$$p(q) \propto e^{-\gamma \mathcal{R}(q)} \tag{14}$$

where $\gamma$ is a constant of order 1. This relationship shows that configurations with remarkably low entropy dynamical responses (i.e., low rattling) are exponentially preferred in the system's steady-state. Thus, rattling and its associated selection principle are able to sufficiently establish the robustness, stability, and persistence of the configurations of complex systems.

In summary, rattling captures the ways in which correlations among disorderly degrees of freedom give rise to system-level fluctuations of different magnitudes. Then, the low-rattling selection principle states that such system-level fluctuations bias the nonequilibrium steady-state of a complex system towards configurations in which the system experiences remarkably low magnitude fluctuations. Furthermore, this spontaneous selection of low rattling configurations necessarily requires that strong correlations between degrees of freedom arise, and thus for orderly behaviors to emerge. Interested readers looking for a complete treatment of this material, as well as theoretical derivations and experimental validation, are referred to [19]. Equipped with a precise way to quantify order in a broad class of complex systems, we may now develop a system-specific understanding of the ways in which symmetry-breaking affects the rattling of our system of beating particles in hopes of finding strategies to stabilize periodic system beating for $N > 2$.

In order to elucidate the role that symmetry-breaking may play in the self-organized states of our system of active microparticles, we must now consider specific system symmetries and their relationship to the magnitude of system-level fluctuations. While our system is not invariant to the action of any obvious continuous symmetry groups, it is permutation-symmetric [24]. This is to say that our collection of microparticles are all dynamically identical (up to fabrication tolerances). Hence, one promising avenue to investigate is the different ways in which permutation-symmetry breaking may lead to order in our system. Based on results from our mechanistic modelling of particle beating, we know that there are two ways in which the dynamics of individual microparticles can be made distinct from one another. First, we know that changing the volumetric

shape of particles will lead to different local hydrodrynamic drag properties. Second, we know that changing the buoyancy of particles also produces local changes to individual microparticle dynamics through its effect on capillary forces. However, changing the shape of our microparticles requires major changes to their fabrication, as well as nontrivial modifications to the mechanistic model. In contrast, we can easily modify a particle's buoyancy by modulating the volume of the bubble forming underneath the particle, which we can in turn control through the size of their Pt patch.

To explore the role of permutation-symmetry breaking on our system, we constructed a simple model that we can work with analytically from the perspective of rattling theory. In line with the rattling ansatz, our model considers the configurational dynamics of collectives of beating particles as a diffusion process. We incorporate the effect of heterogenous particle buoyancies through the inclusion of a parameter modulating the size of bubbles in analogy to the role of the Pt patch. Our beating particles are perfectly suited for this sort of analysis, even more so than others (e.g., robot swarms). In part, this is due to the physics of fluid dynamics at low-Reynolds numbers (∼0.25 Re for our system) [25]. In this regime, inertia ceases to influence the behavior of systems, leaving viscous forces and stochastic thermal fluctuations to affect their dynamics substantially—thereby making a diffusive approximation natural.

Our model elucidates the role of design parameters on the structure of the system-level fluctuations on the basis of two primary assumptions. First, we assume that the behavior of each individual particle $i$ is monotonically modulated by some real-valued design parameter $U_i$ from a set $U = \{U_1, \cdots, U_N\}$ for a system of $N$ particles. These design parameters correspond by analogy to the Pt patch size. Second, we assumed that particle $i$'s bubble burst only affects the other members of the collective and not itself, which broadly matches experimental observations. We can think of the $U_i$ parameters as implicitly determining the strength of the impulse imparted by particle $i$'s bubble burst onto its neighbors. In particular, we model the effect of this parameter and the bubble burst strength $a_i$ according to the following Boltzmann-like monotonic relationship,

$$a_i = \frac{1}{Z} e^{-U_i} \tag{15}$$

where $Z$ is a normalization factor given by $Z = \sum_{i=1}^{N} e^{-U_i}$. In other words, the $a_i$ parameters can be thought of in analogy to the size (and strength) of bubbles that a given particle can support. Hence, we can motivate this modeling choice by envisioning the gas in bubbles distributing itself according to an energy landscape specified by our $U_i$ parameters, and thusly influencing the bubble popping strength $a_i$. The normalization factor $Z$ arises from the fact that we are not interested in the absolute magnitude of the bubble bursts but rather the effect of their relative magnitudes on the collective behavior.

In the main text, we made use of an observable termed the "breathing radius" for purposes of analysis. The breathing radius is the mean Euclidean distance of the particles to the centroid of the collective. Similarly, here we will only consider the statistical properties of the dynamics of a breathing-radius-like observable, $\bar{r}(t)$, under a simple diffusive model. As in the main text, $\bar{r}(t)$ is an averaged quantity over particles: $\bar{r}(t) = \sum_{i=1}^{N} r_i(t)$. By assumption, a bubble burst at particle $i$ leaves particle $i$ stationary, but a burst from some neighbor $j$ exerts an impulse of random direction onto particle $i$. In this case, the dynamics of $r_i(t)$ evolve according to

$$\dot{r}_i(t) = \sum_{j \neq i} a_j \cdot \xi_j \tag{16}$$

where $\xi_j$ is normally-distributed delta-correlated multiplicative noise in the Itô convention. Note that this construction results in an anisotropic diffusion tensor without spatial dependence, as we are not modelling the geometry of interparticle interactions but rather their statistical fluctuations. From this specification of the system's diffusive dynamics, we can apply rattling theory to understand the effect of our design parameters $U_i$ on the self-organized collective behavior of the system.

Given this formulation of the system dynamics, we proceed by calculating the effect of parameter changes on the magnitude of system-level fluctuations. Letting $r(t) = [r_1(t), \cdots, r_N(t)]^T$, the correlation structure of the system is

$$\langle \dot{r}_i(t), \dot{r}_j(t) \rangle = \sum_{k \neq i} \sum_{l \neq j} a_k a_l \delta_{kl} = \sum_{k \neq i,j} a_k^2 = \frac{1}{Z^2} \sum_{k \neq i,j} e^{-2U_k} \tag{17}$$

where $\delta_{kl}$ is the Kronecker delta. We note that the correlation structure of the system has no dependence on time (i.e., it has infinite temporal correlation) and no dependence on configuration $r(t)$, leaving the design parameters $U_i$ as the only variables with an effect on the system behavior. Finally, in order to express the system's rattling in terms of its design parameters we require an analytical expression for the determinant of its covariance tensor, which is challenging in general. Fortunately, for this particular correlation structure there exists such a closed-form expression, which enables us determine the system's rattling as a function of its parameters:

$$\mathcal{R}(U) = \frac{1}{2} \log \det \langle \dot{r}_i(t), \dot{r}_j(t) \rangle = \log \left( \frac{(N-1) \prod_{i=1}^{N} e^{-U_i}}{Z^N} \right). \tag{18}$$

Equipped with an understanding of how the system's parameters affect its rattling (and thus its degree of order), we can now use the model as a tool to guide our experimental design. While there are infinitely many parameter combinations for a

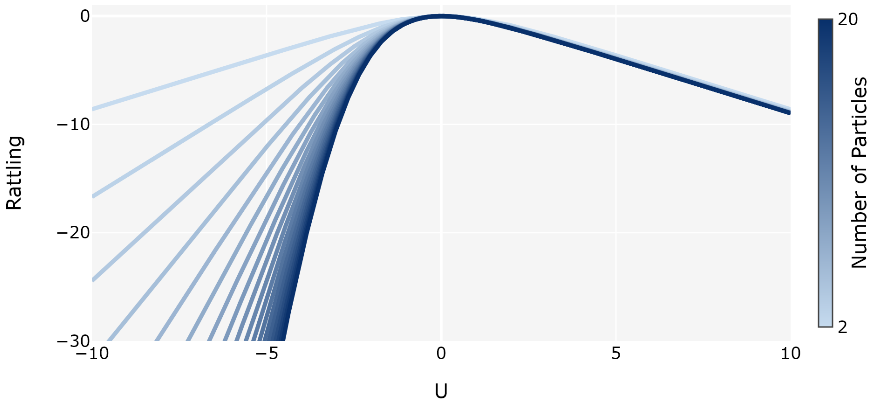


Supplementary Figure 2: **Rattling as a function of patch size in diffusive model.** Here, we study the effect of a given particle's $U$ parameter (in analogy to Pt patch size) on the rattling of collectives of varying sizes. Note that we subtract the constant offset in rattling due to system size so that $\mathcal{R} = 0$ at $U = 0$ for all $N$. We find that any variability in the size of the particle's patch produces a drop in rattling, leading to asymmetry-induced order. When a particle becomes inert as $U$ increases, it stops contributing to system-level fluctuations, leading to a modest drop in rattling independent of $N$. However, as $U$ decreases the modified particle's bubble bursts dominate and effectively become the sole source of variance in the system's configurational degrees of freedom. Such coordination among degrees of freedom leads to a sharp drop in rattling dependent on $N$.

given collection of $N$ particles, one of the simplest design alterations to study is the effect of a single particle differing from the rest—for reasons that we will see shortly, we term this particle a *designated leader*. In this setting, one particle will have its parameter be $U_{DL}$ while the rest of the $N-1$ particles will have it be $\bar{U}$ (which we take to be a constant fixed a priori). Rearranging the expression in Supplementary Equation (18), we have the following expression

$$\mathcal{R}(U_{DL}, N) = -U_{DL} + \log\left(\frac{(N-1)e^{-(N-1)\bar{U}}}{\left(e^{-U_{DL}} + (N-1)e^{-\bar{U}}\right)^N}\right),$$

which allows us to make predictions about the behavior of a collection of $N$ beating particles with a single designated leader.

However, much in the same way that entropy can trivially depend on system size (e.g., number of microstates), our expression for rattling in Supplementary Equation (2) does as well. Thus, to focus on the dependence of $\mathcal{R}(U_{DL}, N)$ on $U_{DL}$, we subtract the constant bias that system size contributes to the value of rattling. To do this, we calculate $\mathcal{R}(U_{DL}, N) - \mathcal{R}(\bar{U}, N)$ for a choice of $\bar{U}$ that we fix across all system sizes, where we note that $\mathcal{R}(\bar{U}, N)$ is merely a constant that offsets the value of rattling to be zero when $U_{DL} = \bar{U}$. Since $\mathcal{R}(\bar{U}, N)$ is exclusively a function of the number of particles for a given $\bar{U}$, subtracting it from $\mathcal{R}(U_{DL}, N)$ precisely removes the constant contribution of system size to the overall magnitude of rattling. As detailed in [19], constant offsets to the rattling values of a system do not affect its behavior. Only changes to the rattling landscape—that is, changes to the relative rattling values between configurations (or parameters)—have an effect on system behavior. This implies that comparing the absolute rattling values across systems is of limited use, which motivates our approach (as in Fig. 2d and Supplementary Fig. 2).

In Supplementary Fig. 2 we show the results of varying the parameters of the designated leader for collectives of various sizes, while fixing $\bar{U} = 0$ and subtracting the bias in rattling due to system size. Crucially, we observe that any deviation from the parameter values of the rest of members of the collective (i.e., away from $U_{DL} = 0$) results in a reduction in rattling. Thus, our model predicts that any amount of heterogeneity will lead to increasingly ordered system states. Such asymmetry-induced order has been studied in networked systems of oscillators [12–15], but its emergence as a low-rattling phenomenon is a novel finding.

Through this mechanism, order arises in one of two distinct ways. First, as $U_{DL}$ increases, the designated leader particle becomes effectively inert. This is to say that the strength of its bubble bursts $a_{DL}$ asymptotically approach zero, as though it were a patchless particle. As a result, the leader particle acts as dead weight and does not contribute to system-level fluctuations, leading to a modest decrease in rattling—independent of the total number of particles—that matches experimental observations. Second, as $U_{DL}$ decreases, the designated leader particle's bubble bursts become stronger and its contribution to the magnitude of system-level fluctuations dominates over those of other particles. In turn, this effectively leads to a concentration of all variability and randomness in the system into a single of its many degrees of freedom, thereby leading to significant correlations in the behavior of all particles and a resulting drop in rattling. Note that as more particles are added more degrees of freedom become correlated, leading to sharper drops in rattling as a function of $N$. Hence, on the basis

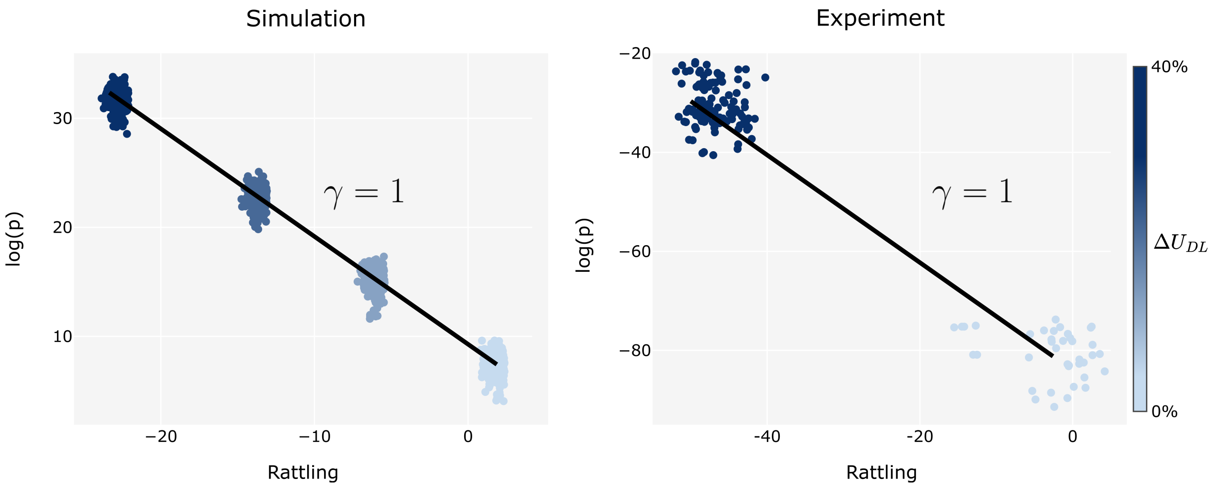


Supplementary Figure 3: **Effect of designated leader on self-organization.** By introducing a designated leader, the entropy of the bubble burst forcing patterns decreases (since they become periodic), which has an effect on the self-organization of the system. On the left panel, we simulate the dynamics in Supplementary Equation (16) and calculate their rattling and steady-state densities numerically. On the right panel, we consider experimental data from an 8 particle collective in both standard ($\Delta U_{DL} = 0\%$) and designated leader configurations ($\Delta U_{DL} = 40\%$), which we then process using the same procedure as for the left panel. While the absolute magnitudes of parameter values for the simulation are arbitrary, the $\Delta U_{DL}$ values are determined from the actual Pt patch sizes used on the experimental systems. For both the simulated and the experimental data, the results are consistent with rattling theory (in particular, Supplementary Equation (19) with $\gamma = 1$). This procedure was then repeated for experimental collectives of other sizes with the same results. Hence, bubble burst patterns of varying entropy (which depend on system design parameters) provide an explanation for the emergence of system order that is consistent with our results.

of these results and other studies of asymmetry-induced order we chose to study the influence of designated leaders on the collective behavior experimentally by producing leader particles with larger Pt patches.

Another consequence of applying the rattling ansatz to the behavior of our collective of beating particles is that it allows one to reinterpret the relationship between system elements. In the theory, configurations with exceptionally orderly responses to external driving forces are selected for the nonequilibrium steady-state of complex systems. Similarly, we can think of the relationship between the particle configurations (i.e., their relative positions and orientations) and the sequence of forces the system experiences due to bubble bursts according to this dichotomy. Working from this perspective, rattling theory then suggests that the entropy of the sequence of bubble bursts can prevent the system from finding orderly configurations by increasing their rattling (see [19], and in particular figure 4 within). More precisely, we have

$$p(q) \propto e^{-\gamma(\mathcal{R}(q)+S(q))}, \tag{19}$$

where $S(q)$ is the entropy of the driving forces affecting the system at configuration $q$. Importantly, this expression shows that the effect of drive entropy is to simply offset a configuration's rattling.

In the main text, we observed that our system design parameters (i.e., the particle Pt patch sizes) do have a profound effect on system behavior and also on the bubble burst sequence—changing its behavior from seemingly random to almost perfectly periodic (see Fig. 2). If we were to accept the hypothesis presented by Supplementary Equation (19), then this difference in behavior should be explained by the difference in the entropy generated by the bubble burst patterns at different system parameters. Moreover, if this is the case, then the results from analyzing standard and designated leader systems should lie on the log-linear correlation of Supplementary Equation (19), with a slope of $\gamma$ (which nominally is of order 1). Indeed, this is precisely what we observe in Supplementary Fig. 3 for simulations of the model dynamics and for our experimental data samples.

While throughout this section we have motivated the analytical model in specific reference to our experimental beating particles, our model and results generalize beyond our system. At its core, our generic model describes the structure of statistical fluctuations in a collection of strongly interacting degrees of freedom (i.e., under strong mixing conditions). This is to say that the details of how the magnitudes of said fluctuations are parametrized by system properties are inessential to the results. Particularly, the equation for rattling in Supplementary Equation (18) can be expressed in terms of $a_i$ directly as $\log\left((N-1)\prod_{i=1}^{N} a_i\right)$, which allows one to freely model the way in which individual degrees of freedom contribute to the overall system-level fluctuations. Thus, rattling theory, as well as the mechanism we have outlined for asymmetry-induced

order, present a general framework from which to understand the effect of system design parameters on the self-organized behaviors of the system—providing a novel approach to micro-system design based on thermodynamic principles relevant to the scales of interest.

## 3 Note on Microelectronic Low-Frequency Oscillators

In this section, we elaborate on the design and fabrication challenges of microelectronic oscillators with a frequency on the order of a hertz, which we briefly alluded to in the Introduction part of the main text. Given the relatively large footprints of integrated capacitors and inductors available, RC- and LC-based oscillators are hardly compatible with the limited space on micrometre-sized machines [26]. For example, the frequencies of RC oscillators, such as a bi-inverter or a Schmidt Trigger oscillator, are on the order of the reciprocal of their respective RC constants, i.e. $f_{\mathrm{RC}} \sim \mathcal{O}(1/RC)$. Taking the capacitance to be a generous 40pF for an area of $100\mu$m$\times 100\mu$m [27], one would require a massive resistor of 25G$\Omega$ to achieve an RC time constant of 1s. Assuming a resistivity of 100k$\Omega/\mu$m$^2$ of polysilicon, this resistor alone would occupy $2.5\times 10^5\mu$m$^2$. Alternatively, one may opt to use a frequency divider to bring the kHz-order frequency of a typical microelectronic relaxation oscillator down to 1Hz. Suppose the starting frequency is 17kHz [28], a cascade of 15 T flip-flops is needed, each of which is constructed from at least 20 transistors [29]. Should 300 transistors be fabricated onto a $100\mu$m$\times 100\mu$m microchip, the appropriate transistor node would be 500nm. While well within the realm of possibility, such technology typically still requires the involvement of a commercial foundry outside of academic institutions. Similarly, thyristor-based oscillators of frequencies from 20Hz and up have been foundry-fabricated with a feature size of 180nm [26]. The integrated circuit design expertise and capital investment required are the reasons for a high barrier-to-entry. Note that the area reserved for onboard energy harvesting and storage units, as well as for miscellaneous electronics, may further constrain the real estate available to the microelectronic oscillator.

## 4 Note on the Fuel Cell's Open-Circuit Voltage

Supplementary Fig. 13 shows that the open-circuit voltages of the Pt-Au and Pt-Ru fuel cell devices, $V_{\mathrm{OC}}$, exhibits a very weak dependence on the peroxide concentration $[H_2O_2]$, unlike the trend of the short-circuit current densities (Fig. 4c and Supplementary Fig. 14). Here we provide a simple explanation based on electrochemical kinetics. We consider the following two pairs of forward and reverse reactions taking place on a single electrode:

$$\left.\begin{array}{l} \mathrm{H_2O_2} \longrightarrow \mathrm{O_2} + 2\mathrm{H^+} + 2\mathrm{e^-} \\[1em] \mathrm{O_2} + 2\mathrm{H^+} + 2\mathrm{e^-} \longrightarrow \mathrm{H_2O_2} \end{array}\right\} \mathrm{R_1},\ \phi^{\circ}_{\mathrm{eq},1} = +0.68\mathrm{V}$$

$$\left.\begin{array}{l} 2\mathrm{H_2O} \longrightarrow \mathrm{H_2O_2} + 2\mathrm{H^+} + 2\mathrm{e^-} \\[1em] \mathrm{H_2O_2} + 2\mathrm{H^+} + 2\mathrm{e^-} \longrightarrow 2\mathrm{H_2O} \end{array}\right\} \mathrm{R_2},\ \phi^{\circ}_{\mathrm{eq},2} = +1.77\mathrm{V}$$

where $\phi^{\circ}_{\mathrm{eq}}$ denotes the standard equilibrium potentials. The Butler–Volmer equation suggests that only one half-reaction from $R_1$ and $R_2$ each is dominant at the mixed potential $\phi_{\mathrm{mix}}$, defined as the potential where the total current equals 0 [30]. If we consider the oxidative half-reaction of $R_1$ and the reductive half-reaction of $R_2$ (choosing the other two half-reactions does not alter the conclusion), the full Butler-Volmer kinetic expression is given by [31]:

$$i_1(\phi) = nFk_1[\mathrm{H_2O_2}]^{\nu_{\mathrm{H_2O_2}}} \exp\left[\frac{\alpha_1 F}{\bar{R}T}\phi\right] \tag{20}$$

$$i_2(\phi) = -nFk_2[\mathrm{H_2O_2}]^{\nu_{\mathrm{H_2O_2}}}[\mathrm{H^+}]^{\nu_{\mathrm{H}}} \exp\left[-\frac{(1-\alpha_2)F}{\bar{R}T}\phi\right] \tag{21}$$

where $\phi$ is the applied potential on the absolute scale, $i_1(\phi)$ and $i_2(\phi)$ the respective current densities, $n$ the number of electrons transferred, $F$ the Faraday constant, $k$ the rate constants, $\nu$ the reaction orders, $\alpha$ the the transfer coefficients, $\bar{R}$ the universal gas constant, $T$ the absolute temperature. We can obtain the mixed potential $\phi_{\mathrm{mix}}$ by solving:

$$i_1(\phi_{\mathrm{mix}}) + i_2(\phi_{\mathrm{mix}}) = 0 \tag{22}$$

which is equivalent to $-i_1(\phi_{\mathrm{mix}})/i_2(\phi_{\mathrm{mix}}) = 1$. While the exact form of the solution is of little relevance to us, the division of the right-hand side of Supplementary Equation (20) by that of Supplementary Equation (21) reveals the cancellation of the $[H_2O_2]$ terms under the typical assumption of equal reaction order. That is, $\phi_{\mathrm{mix}}$ is independent of the peroxide concentration for a given electrode. Because the open-circuit voltage between two spatially separated electrodes (such as Pt and Ru) is essentially the difference in the respective mixed potentials ($\Delta\phi_{\mathrm{mix}}$), $V_{\mathrm{OC}}$ naturally sees little dependence on $[H_2O_2]$. This allows us to compare our $V_{\mathrm{OC}}$ measurements with past mixed potential studies carried out at lower $[H_2O_2]$. For example, Wang and colleagues [32] measured a $\Delta\phi_{\mathrm{mix}}$ of 30mV between Pt and Au, and 140mV between Pt and Ru, both consistent with our results.

## 5 Note on the Energy Expenditure

### 5.1 Energy Conversions of the Mechanical Oscillation

Within each period of the emergent mechanical oscillation, chemical energy stored in the $H_2O_2$ fuel is converted into the particles' kinetic energy upon the collapse of the $O_2$ bubble. The kinetic energy imparted to two outgoing particles simply take the form of $E_k = mv^2$, where $m$ is the mass of each particle and $v$ the maximal velocity right following the bubble collapse. With $m = 2.34\mu$g for a $500\mu$m-diameter particle and $v = 3.2 \times 10^4\mu$m/s measured from experiments, $E_{out}$ is estimated to be $2.40 \times 10^{-12}$J per cycle.

The chemical energy consumed per cycle may be computed as:

$$E_{chem} = \frac{2PV_{b,th}\Delta H}{RT}$$

where $V_{b,th}$ denotes the bubble volume at threshold, estimated to be $9.81\times10^{-2}\mu$L in a 2-particle homogeneous system in 1mL of 10% $H_2O_2$. We assume an ambient pressure $P$ of 1atm and temperature $T$ of 25°C, as the excess Laplace pressure within the bubble before collapse is a negligible $5.0 \times 10^{-3}$atm. $\Delta H$, the enthalpy change of the decomposition reaction, is 98.24kJ/mol at given conditions, equivalent to an energy density of 2.89kJ/g $H_2O_2$ or 0.29kJ/g 10wt% $H_2O_2$ solution [33]. $E_{chem}$ per cycle is computed to be $7.88 \times 10^{-4}$J. The portion of the chemical energy converted to the work of expansion is:

$$W_{PV} = P_{atm}V_{b,th} + 4\pi\gamma R_{b,th}^2$$

where $R_{b,th}$ is the threshold radius assuming a spherical bubble. The latter term of $7.41 \times 10^{-8}$J is the surface energy $E_{surf}$, i.e. the work against the Laplace pressure during bubble growth. To summarize, therefore, 1.26% of the original chemical energy contributes to a $W_{PV}$ of $1.00 \times 10^{-5}$J. 0.74% of the work of expansion is stored as the surface energy. Finally, the kinetic energy gained by the particles account for 0.032‰ of surface energy stored in the bubble.

### 5.2 Energy Conversions of the Microgenerators

As the microgenerator converts the chemical energy from $H_2O_2$ decomposition to electrical work, it is of interest to calculate the proportion of total $H_2O_2$ molecules consumed which contributed to the electrical current [34, 35]. Given that each electrochemically redoxed $H_2O_2$ molecule transfers an electron, ON-state currents of 180.66nA (in the absence of an electrical load) and 15.27nA (with a load, i.e. the actuator) are respectively attributed to $1.87\times10^{-12}$ and $1.58\times10^{-13}$ moles of $H_2O_2$ per second. These correspond to 0.76‰ and 0.063‰ of the total peroxide consumption rate ($2P/RT \cdot dV_b/dt = 2.45 \times 10^{-9}$mol/s), respectively. The former is in agreement with prior literature [34], which estimated an electrochemical contribution of 0.5‰. Since more than 99.9% of the consumed $H_2O_2$ decompose via the same non-electrochemical pathway as in the beating particles with no fuel cells aboard, generation of the electrical current has a negligible impact on the mechanical oscillation if all other conditions are kept the same. Along the same lines, additional fuel cell particles are not expected to diminish the electrical signals observed.

## Supplementary Figures

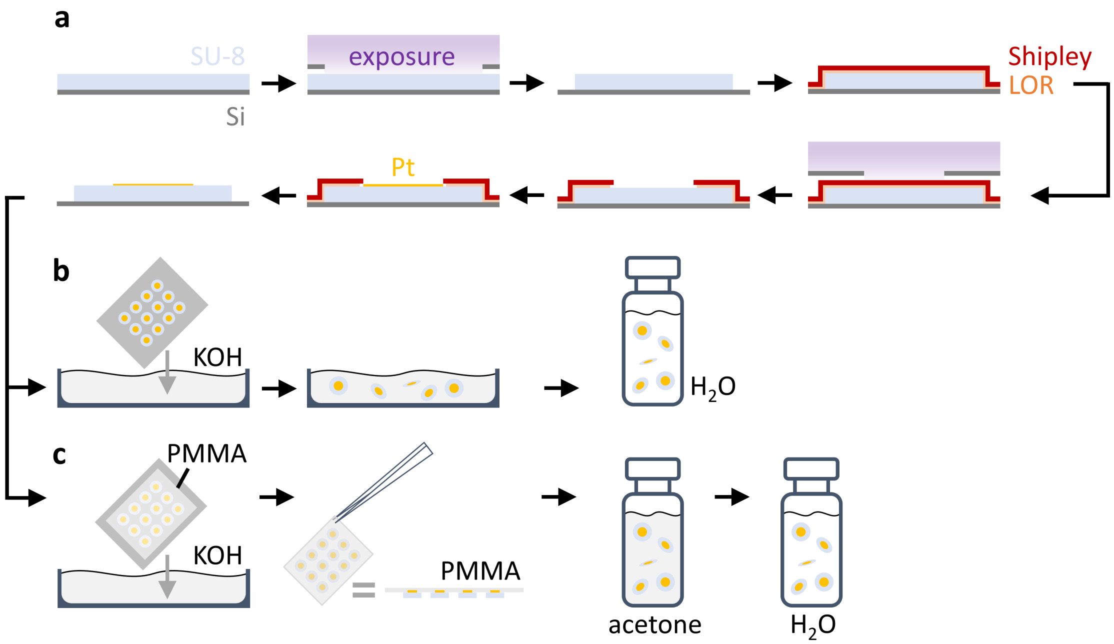


Supplementary Figure 4: **Beating particle fabrication steps.** **a**, An array of SU-8 polymeric microdiscs were defined and patterned on a Si wafer with standard photolithography, followed by electron-beam physical vapor deposition of Pt on top. **b**, The particles were subsequently lifted off in heated KOH solution which etched into the Si substrate. The KOH was displaced by water in which the lifted off microparticles were stored. **c**, Alternatively, a film of PMMA polymer was spun over the microparticle array. Together they would delaminate from the substrate in heated KOH solution. The PMMA was then removed with an acetone rinse. The lifted off particles were transferred to water for storage.

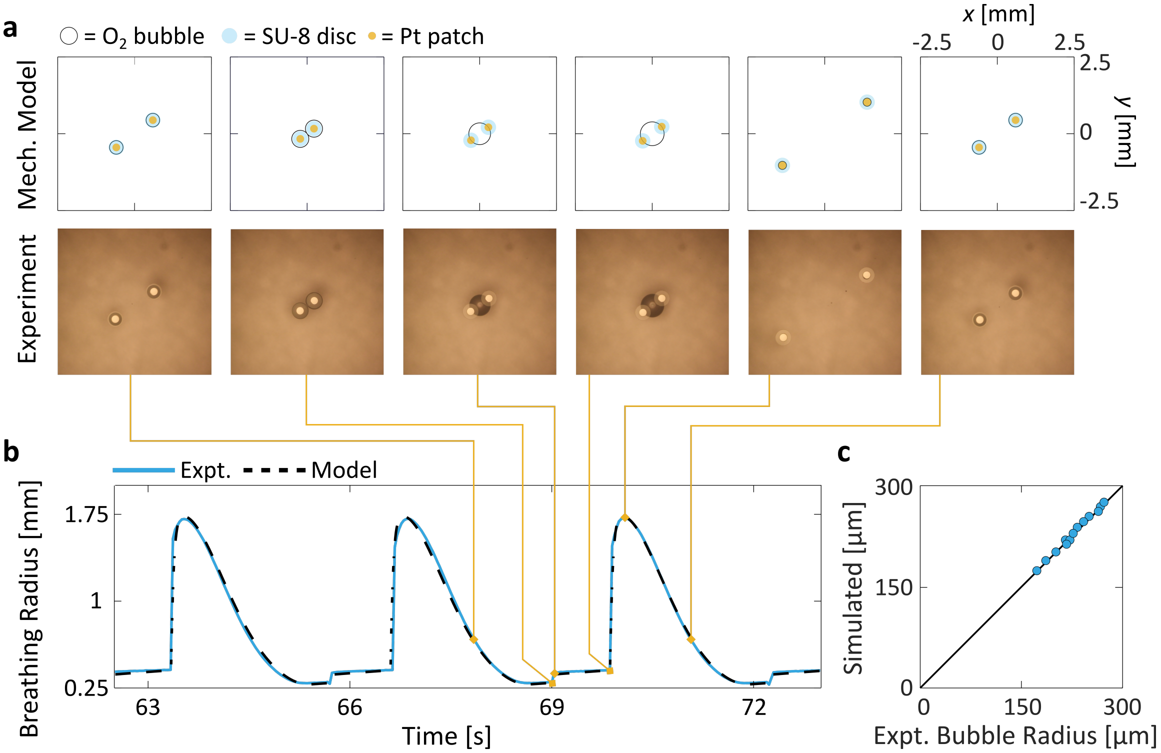


Supplementary Figure 5: **Detailed comparison between experimental and simulated beating behaviours of two particles. a,** Mechanistic model simulations and experimental snapshots taken at representative stages of a beating cycle. **b,** The simulation and experiments are in excellent agreement, evident from the matching curves of the breathing radius, previously also shown in Fig. 1g. We note that the mechanistic model captures fine details of the self-oscillation, such as the subtle step change in (**b**) at approximately 69s. The step increase was a result of the merged bubble pushing the particles outwards slightly, reflected by both the experiment and simulation in (**a**). **c,** This panel shows the excellent agreement between the experimental bubble radii and those predicted by the mechanistic model. The former were measured manually from the raw video data.

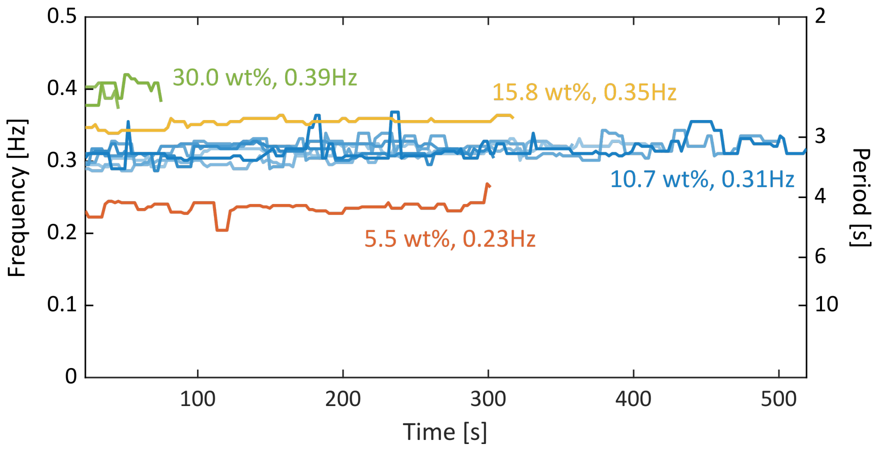


Supplementary Figure 6: **Beating frequencies over time from moving window recurrence analyses.** The same histograms as in Fig. 1i of the main text were generated, but here only for breathing radius data within a moving window of 150 frames (5s). Frequencies calculated from the most probable recurrence time of each window were plotted as a function of time. The beating frequencies in all experiments are constant throughout, demonstrating robust periodicity. Furthermore, curves from experimental replicates overlap. The frequencies from moving window analyses agree with those shown in Fig. 1j for all the $H_2O_2$ concentrations. These concentrations respectively correspond to 6-, 3-, 2-, and 1-fold volumetric dilution of a 30wt% $H_2O_2$ solution.

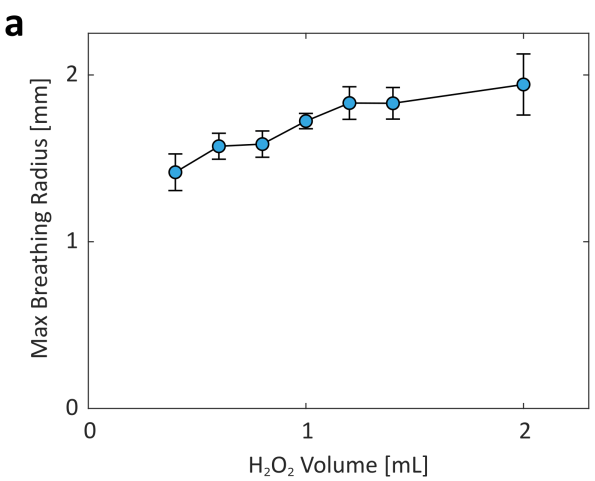


Supplementary Figure 7: **Maximum breathing radius and interarrival time of two identical particles as a function of the $H_2O_2$ volume.** A larger volume of $H_2O_2$ solution corresponds to a reduced curvature of the liquid-air interface the particles reside in, which in turn weakens the global restorational force that resists parting of the particles. The breathing radius (**a**) therefore increases with the $H_2O_2$ volume, which consequently lengthens the intervals between consecutive bubble collapses (**b**). Due to the periodicity of all these 2-particle systems, the respective interarrival times are equivalent to the periods of oscillation. Each error bar denotes a standard deviation among the oscillation cycles within an experiment.

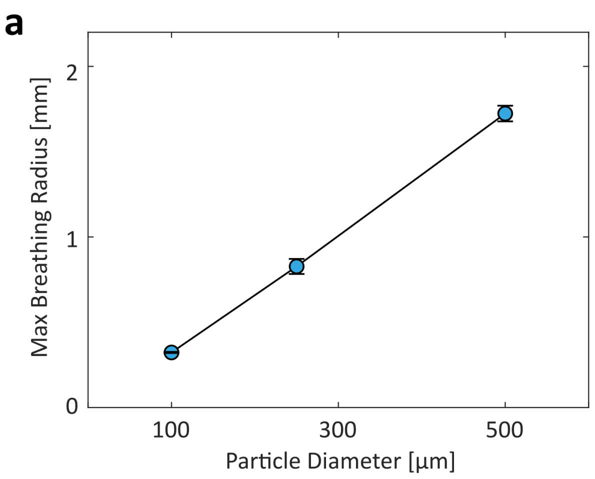


Supplementary Figure 8: **Maximum breathing radius and interarrival time of two identical particles as a function of the particle size.** All particles were fabricated by depositing 5nm Cr and 50nm Pt onto 10$\mu$m-thick SU-8 polymer. The 500$\mu$m, 250$\mu$m, and 100$\mu$m-diameter particles were designed to have Pt patches 250$\mu$m, 125$\mu$m, and 100$\mu$m in diameter, respectively. As with Supplementary Figure 7, the oscillation amplitude exhibits an increasing trend with respect to the particle diameters (**a**). The interarrival times (**b**), however, decrease with the particle size. Due to the periodicity of all these 2-particle systems, the respective interarrival times are equivalent to the periods of oscillation. Each error bar denotes a standard deviation among the oscillation cycles within an experiment.

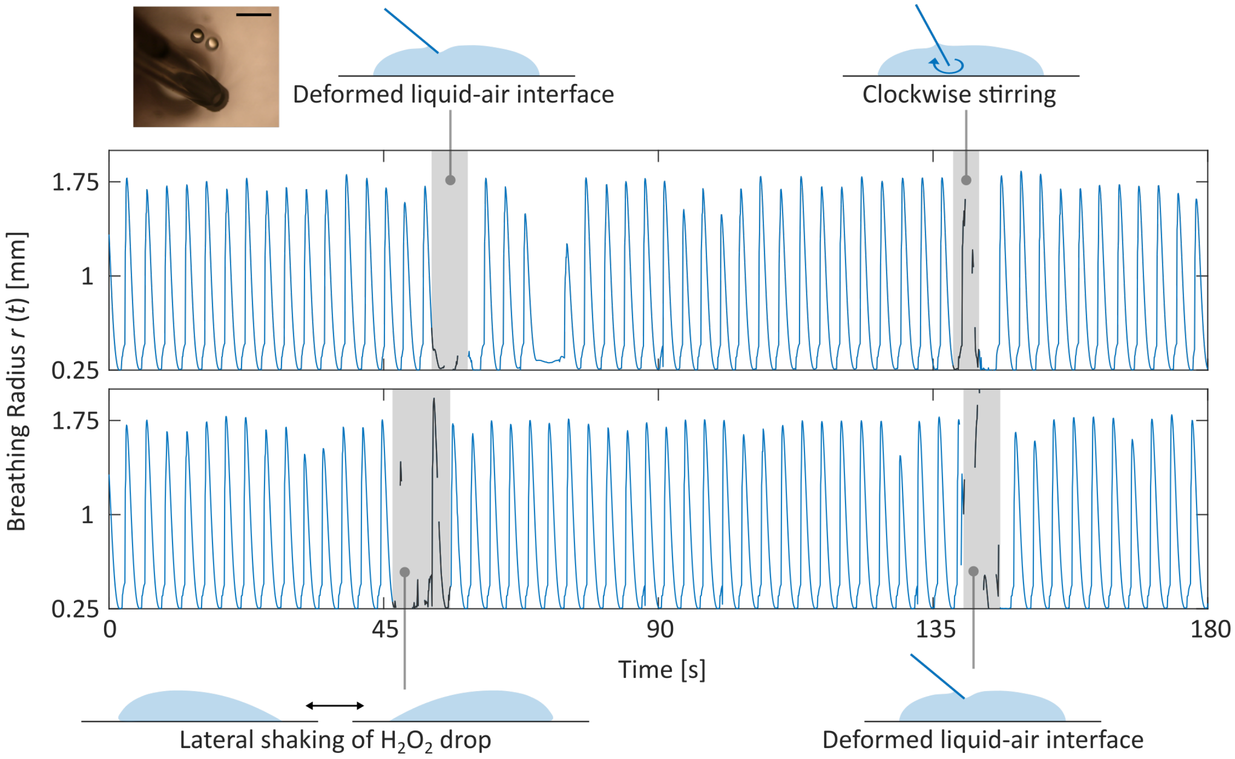


Supplementary Figure 9: **Robustness of the emergent oscillation to perturbations.** In these two experiments, we intentionally disturbed a system of two identical particles by (i) deforming the liquid-air interface with a pipette [36], (ii) stirring the $H_2O_2$ drop, and (iii) shaking the drop back and forth. It is evident in the breathing radius trajectories that the collective oscillation resumes promptly following the perturbations (shaded region) with its amplitude and periodicity unchanged, thus demonstrating robustness. Data discontinuities during the perturbations are a result of blurry frames or particles temporarily exiting the camera field-of-view. The inset micrograph shows the particles approaching the pipette due to the deformed interface. Scale bar, 1mm.

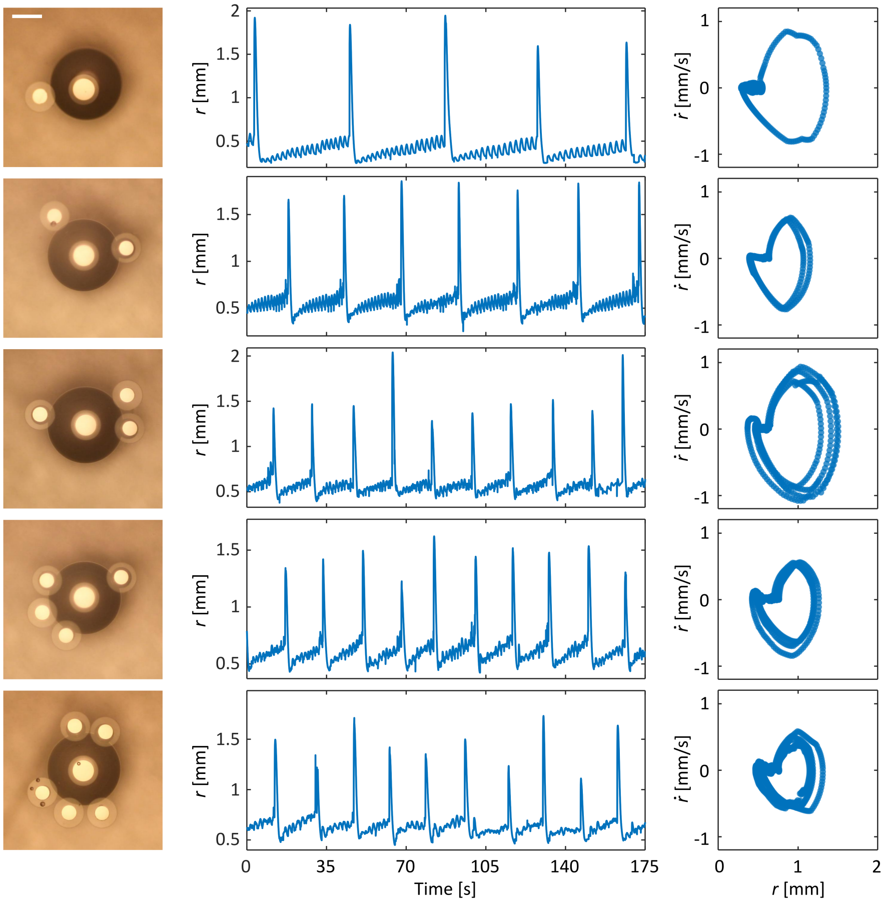


Supplementary Figure 10: **Compiled snapshots, breathing radius trajectories, and phase portraits for heterogeneous/DL systems of $N$ = 2 to 6.** Systems of all sizes exhibited clear periodicity in their beating behaviours with stable limit cycles. Scale bar, 500$\mu$m. All experiments were performed in 1mL of 10.7wt% $H_2O_2$.

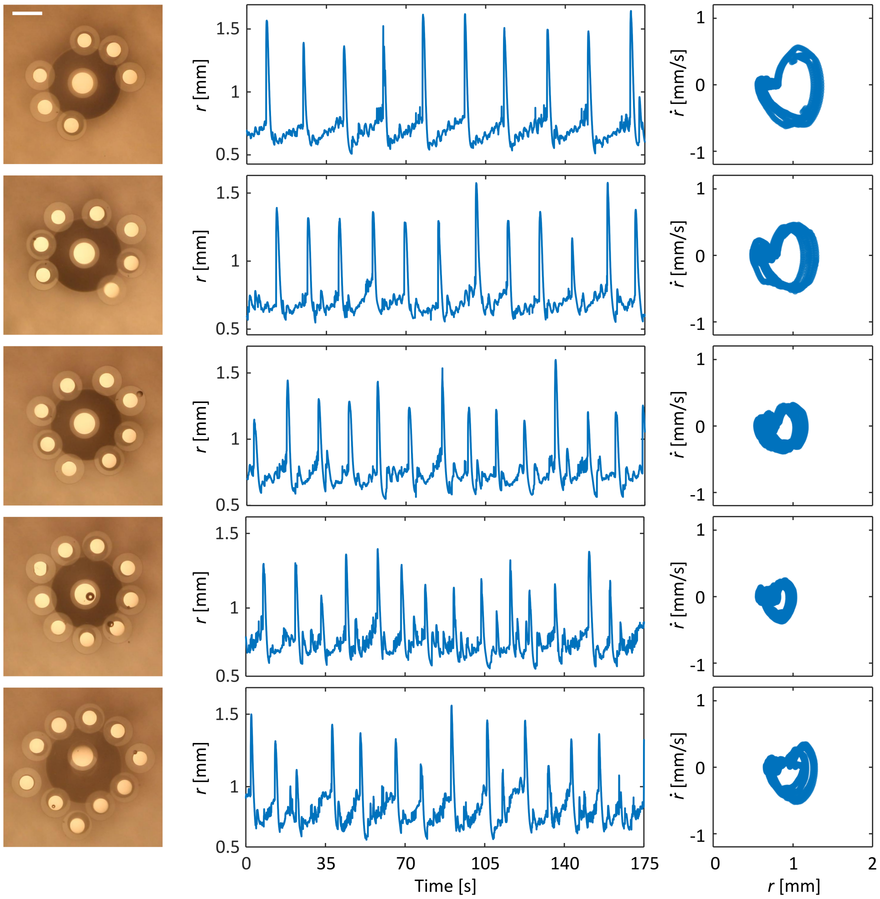


Supplementary Figure 11: **Compiled snapshots, breathing radius trajectories, and phase portraits for heterogeneous/DL systems of $N$ = 7 to 11.** Systems of all sizes exhibited clear periodicity in their beating behaviours with stable limit cycles. Scale bar, 500$\mu$m. All experiments were performed in 1mL of 10.7wt% $H_2O_2$.

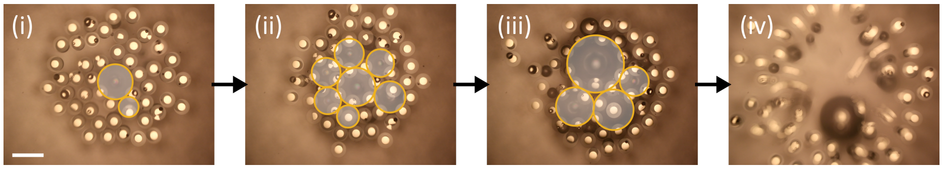


Supplementary Figure 12: **Progression of a large-scale homogeneous collection.** While we have established in Fig. 2 of the main text that the periodicity of homogeneous systems breaks down easily as $N$ increases, we observe intriguing hierarchical organization of the bubbles in this 50-particle collective over a span of 8s. Bubbles from individual particles merge and grow (i), resulting in the intermediate situation in (ii) where a large bubble situated at the $H_2O_2$ drop's apex is packed around by smaller ones. Following further merger and growth (iii), the system eventually collapses (iv). Highlighted in yellow circles are bubbles larger than 350$\mu$m in radius. With a number of particles distributed along the perimeter, a bubble is observed to grow far beyond the typical threshold size in few-particle homogeneous systems (*cf.* bubble sizes in DL systems in Supplementary Figs. 10 and 11). Scale bar, 1mm. All experiments were performed in 1mL of 10.7wt% $H_2O_2$.

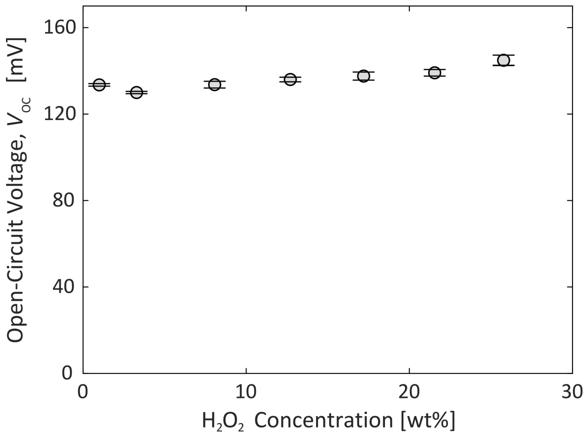


Supplementary Figure 13: **Open-circuit voltage of a Pt-Ru device as a weak function of $H_2O_2$ concentration.** The observation is explained by the auto-redox nature of the $H_2O_2$ decomposition reaction (Supplementary Section 4). Error bar, standard deviation.

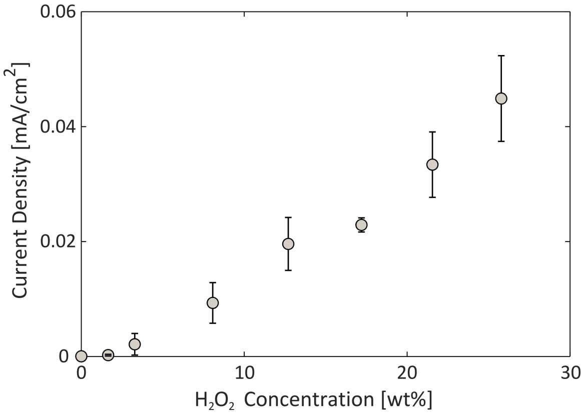


Supplementary Figure 14: **Short-circuit current density as a function of $H_2O_2$ concentration for a Pt-Au device.** (*cf.* Fig. 4c of the main text). Error bar, standard deviation.

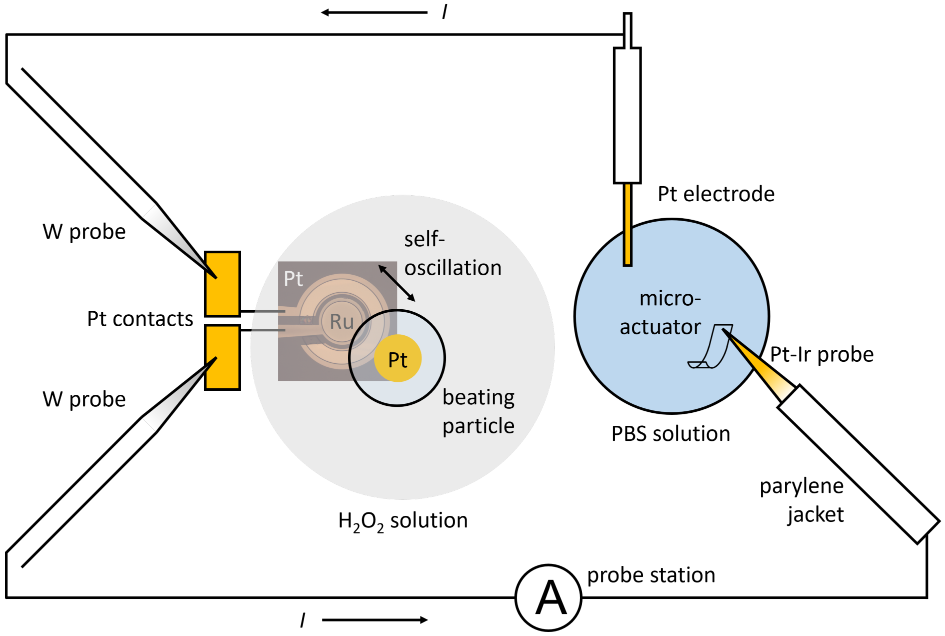


Supplementary Figure 15: **Bimorph actuator experimental setup.** The microactuator in PBS solution is connected via external wiring to the beating system in an $H_2O_2$ drop. The mechanical self-oscillation is translated to an oscillatory electrical current as illustrated in Fig. 4a, which powers cyclic motion of the actuator (Fig. 4e).

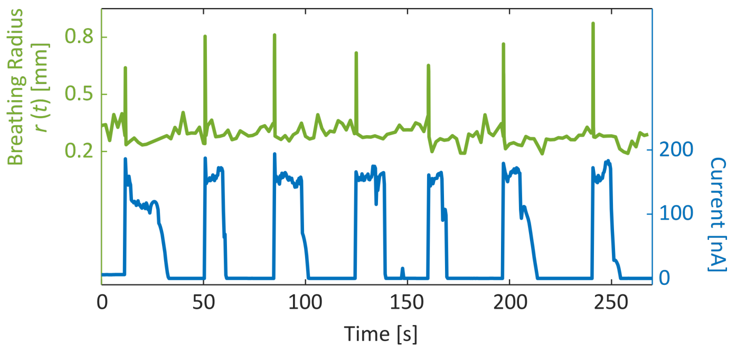


Supplementary Figure 16: **Oscillatory mechanical beating drives on-board oscillatory current.** (See also Fig. 4e of the main text). As a standard 500-$\mu$m particle beats with a Pt-Ru fuel cell device (Fig. 4b, also Methods), the bubbles collapse at regular intervals as indicated by the spikes in the breathing radius trajectory ($r(t)$, top). Removal of the bubbles restores the electrochemical reactivity of the fuel cell electrodes, and therefore the current (bottom) peaks precisely as $r(t)$ does. The current measured in this experiment is an order of magnitude higher than that in Fig. 4e since the system characterized here was not connected to an actuator.

## Supplementary References


1. Del Río, O. I. & Neumann, A. W. Axisymmetric drop shape analysis: Computational methods for the measurement of interfacial properties from the shape and dimensions of pendant and sessile drops. *Journal of Colloid and Interface Science* **196,** 136–147. ISSN: 00219797 (1997).
2. Hauser, A. W., Sundaram, S. & Hayward, R. C. Photothermocapillary oscillators. *Physical Review Letters* **121,** 158001. ISSN: 0031-9007 (Oct. 2018).
3. Nicolson, M. M. The interaction between floating particles. *Mathematical Proceedings of the Cambridge Philosophical Society* **45,** 288–295. ISSN: 14698064 (1949).
4. Vella, D. & Mahadevan, L. The "Cheerios effect". *American Journal of Physics* **73,** 817–825 (2005).
5. Kralchevsky, P. A. & Nagayama, K. Capillary interactions between particles bound to interfaces, liquid films and biomembranes. *Advances in Colloid and Interface Science* **85,** 145–192. ISSN: 00018686 (2000).
6. Dalbe, M.-J., Cosic, D., Berhanu, M. & Kudrolli, A. Aggregation of frictional particles due to capillary attraction. *Physical Review E* **83,** 051403. ISSN: 1539-3755. arXiv: 1104.3820 (May 2011).
7. Das, S., Koplik, J., Somasundaran, P. & Maldarelli, C. Pairwise hydrodynamic interactions of spherical colloids at a gas-liquid interface. *Journal of Fluid Mechanics* **915,** A99. ISSN: 0022-1120 (May 2021).
8. Batchelor, G. K. Brownian diffusion of particles with hydrodynamic interaction. *Journal of Fluid Mechanics* **74,** 1–29. ISSN: 14697645 (1976).
9. Vassileva, N. D., Van Den Ende, D., Mugele, F. & Mellema, J. Capillary forces between spherical particles floating at a liquid-liquid interface. *Langmuir* **21,** 11190–11200. ISSN: 07437463 (2005).
10. Plauck, A., Stangland, E. E., Dumesic, J. A. & Mavrikakis, M. Active sites and mechanisms for $H_2O_2$ decomposition over Pd catalysts. *Proceedings of the National Academy of Sciences* **113,** E1973–E1982 (2016).
11. Lin, S.-S. & Gurol, M. D. Catalytic Decomposition of Hydrogen Peroxide on Iron Oxide: Kinetics, Mechanism, and Implications. *Environmental Science & Technology* **32,** 1417–1423. ISSN: 0013-936X (1998).
12. Medeiros, E. S., Feudel, U. & Zakharova, A. Asymmetry-induced order in multilayer networks. *Phys. Rev. E* **104,** 024302 (2 2021).
13. Nicolaou, Z. G., Case, D. J., Wee, E. B. v. d., Driscoll, M. M. & Motter, A. E. Heterogeneity-stabilized homogeneous states in driven media. *Nature Communications* **12,** 4486. ISSN: 2041-1723 (2021).
14. Zhang, Y., Ocampo-Espindola, J. L., Kiss, I. Z. & Motter, A. E. Random heterogeneity outperforms design in network synchronization. *Proceedings of the National Academy of Sciences* **118.** ISSN: 0027-8424 (2021).
15. Zhang, Y., Nishikawa, T. & Motter, A. E. Asymmetry-induced synchronization in oscillator networks. *Phys. Rev. E* **95,** 062215 (6 2017).
16. Landauer, R. Inadequacy of entropy and entropy derivatives in characterizing the steady state. *Phys. Rev. A* **12,** 636–638 (2 1975).
17. Vicsek, T., Czirók, A., Ben-Jacob, E., Cohen, I. & Shochet, O. Novel Type of Phase Transition in a System of Self-Driven Particles. *Phys. Rev. Lett.* **75,** 1226–1229 (6 Aug. 1995).
18. Parisi, G. Order parameter for spin-glasses. *Physical Review Letters* **50,** 1946 (1983).
19. Chvykov, P. *et al.* Low rattling: A predictive principle for self-organization in active collectives. *Science* **371,** 90–95. ISSN: 0036-8075 (2021).
20. Chvykov, P. & England, J. Least-rattling feedback from strong time-scale separation. *Phys. Rev. E* **97,** 032115 (3 2018).
21. Jackson, Z. & Wiesenfeld, K. Emergent, linked traits of fluctuation feedback systems. *Phys. Rev. E* **104,** 064216 (6 2021).
22. Palacci, J., Sacanna, S., Steinberg, A. P., Pine, D. J. & Chaikin, P. M. Living Crystals of Light-Activated Colloidal Surfers. *Science* **339,** 936–940. ISSN: 0036-8075 (2013).
23. Corte, L., Chaikin, P., Gollub, J. P. & Pine, D. Random organization in periodically driven systems. *Nature Physics* **4,** 420–424 (2008).
24. Ferré, G., Maillet, J.-B. & Stoltz, G. Permutation-invariant distance between atomic configurations. *The Journal of Chemical Physics* **143,** 104114 (2015).
25. Purcell, E. M. Life at low Reynolds number. *American Journal of Physics* **45,** 3–11 (1977).
26. Funke, D. A. *et al.* Ultra low-power,-area and-frequency CMOS thyristor based oscillator for autonomous microsystems. *Analog Integrated Circuits and Signal Processing* **89,** 347–356 (2016).

27. Molnar, A. C. *et al. Nanoliter-scale autonomous electronics: Advances, challenges, and opportunities* in *2021 IEEE Custom Integrated Circuits Conference (CICC)* (IEEE, Apr. 2021), 1–6. ISBN: 978-1-7281-7581-2.

28. Lee, S. *et al.* A 250 $\mu$m × 57 $\mu$m microscale opto-electronically transduced electrodes (MOTEs) for neural recording. *IEEE Transactions on Biomedical Circuits and Systems* **12,** 1256–1266. ISSN: 1932-4545. https://ieeexplore.ieee.org/document/8491379/ (Dec. 2018).

29. Harris, S. L. & Harris, D. *Digital Design and Computer Architecture* RISC-V. ISBN: 9780128200643 (Elsevier Inc., 2021).

30. Park, J. H. *et al.* Open circuit (mixed) potential changes upon contact between different inert electrodes-size and kinetic effects. *Analytical Chemistry* **85,** 964–970. ISSN: 00032700 (2013).

31. Corbin, N., Zeng, J., Williams, K. & Manthiram, K. Heterogeneous molecular catalysts for electrocatalytic $CO_2$ reduction. *Nano Research* **12,** 2093–2125. ISSN: 1998-0124 (Sept. 2019).

32. Wang, Y. *et al.* Bipolar electrochemical mechanism for the propulsion of catalytic nanomotors in hydrogen peroxide solutions. *Langmuir* **22,** 10451–10456. ISSN: 07437463 (2006).

33. Wehner, M. *et al.* An integrated design and fabrication strategy for entirely soft, autonomous robots. *Nature* **536,** 451–455 (2016).

34. Wang, W., Chiang, T.-y., Velegol, D. & Mallouk, T. E. Understanding the efficiency of autonomous nano- and microscale motors. *Journal of the American Chemical Society* **135,** 10557–10565 (July 2013).

35. Paxton, W. F. *et al.* Catalytically Induced Electrokinetics for Motors and Micropumps. *Journal of the American Chemical Society* **128,** 14881–14888 (2006).

36. Solovev, A. A., Mei, Y. & Schmidt, O. G. Catalytic Microstrider at the Air–Liquid Interface. *Advanced Materials* **22,** 4340–4344 (2010).
