# Supplementary material for: Emergent microrobotic oscillators via asymmetry-induced order
Source: Nat Commun. 2022 Oct 13;13:5734. doi: 10.1038/s41467-022-33396-5 (PMC9561614; doi:10.1038/s41467-022-33396-5)
Supplement: Supplementary file 3 — Description of Additional Supplementary Files [file 41467_2022_33396_MOESM3_ESM.pdf]

Description of Additional Supplementary Files

Supplementary Movie 1: **Motionless single-particle system (*N* = 1).** Initially, the bubble underneath quickly grows due to the catalytic decomposition of H_2O_2. The gradual blockage of the Pt patch by the growing bubble limits the catalyst's access to the fuel asymptotically. The bubble therefore remains smaller than the critical size of collapse for a prolonged time.

Supplementary Movie 2: **Emergent periodic beating of two identical particles (*N* = 2).** The previously self-limiting condition in Supplementary Movie 1 is liberated as two bubbles contact and coalesce. The catalytic surfaces freed up from blockage resumes O_2 generation, eventually leading to a bubble size beyond the threshold. The bubble's collapse resets the system and starts the next cycle of collective oscillation. The breathing radius r as a function of time *t* is shown at the bottom left corner. Downsampled by a factor of 5 from the raw data.

Supplementary Movie 3: **Periodic beating of a 2-particle heterogeneous system with a "designated leader" (*N* = 1 + 1DL).** Identical to the standard particles in terms of geometry and mass, the designated leader is distinct only in the enlarged Pt patch. The heterogeneity among the O_2 generation rates result in a modified beating behaviour of a significantly lower frequency. The breathing radius $r(t)$ is shown at the bottom left corner. Downsampled by a factor of 5 from the raw data.

Supplementary Movie 4: **Periodicity surprisingly retained in a heterogeneous system of 8 particles, stabilized by the designated leader via asymmetry-induced order (*N* = 7 + 1DL).** At large system sizes, robust chemomechanical oscillation is only attainable through intentionally breaking the permutation symmetry, evident from the contrast between this video and the random-process-like behaviour among 8 identical particles (Figs. 2b and c). The breathing radius $r(t)$ is shown at the bottom left corner. Downsampled by a factor of 5 from the raw data.

Supplementary Movie 5: **The cyclic actuation of a microrobotic arm driven by the on-board oscillatory electrical current.** Illustrated in Fig. 4a, we translated the chemomechanical beating to a usable periodic electrical signal via a simple on-board bimetallic fuel cell. This current actuates the tip of the microrobotic arm repeatedly with minimal phase delay, shown in the time series data of the measured current (I) and the actuator length change (ΔLength). The video was sharpened with the unsharp masking technique and flat-field corrected. It was sped up 35 times.
